# Deterioration of diabetic nephropathy via stimulating secretion of cytokines by atrial natriuretic peptide

**DOI:** 10.1186/s12902-021-00867-7

**Published:** 2021-10-18

**Authors:** Chenxiao Liu, Qi Li, Xiu Feng, Jian Zhu, Qian Li

**Affiliations:** 1 Department of Endocrinology, Suzhou Municipal Hospital, Nanjing Medical University, 242 Guangji Road, Suzhou, China; 2 Department of Endocrinology, Nanjing First Hospital, Nanjing Medical University, 68 Changle Road, Qinhuai District, Nanjing, 210006 Jiangsu Province China; 3 Department of Endocrinology, The First People’s Hospital of Changzhou, The Third Affiliated Hospital of Soochow University, Changzhou, China

**Keywords:** Atrial natriuretic peptide, Type 2 diabetes, Diabetic nephropathy, Albuminuria, Cytokines

## Abstract

**Background:**

Atrial natriuretic peptide (ANP) is a cardiovascular and metabolic hormone that has been identified recently as being associated with chronic kidney disease (CKD) without diabetes. Cytokines such as interleukin-6 (IL-6), tumor necrosis factor-α (TNF-α) and adiponectin (ADP) contribute to the development of type 2 diabetes (T2DM). The aim here was to investigate the relationships of ANP with cytokine levels and clinical variables in T2DM nephropathy patients.

**Methods:**

A total of 81 participants with T2DM were recruited, including 37 patients with normoalbuminuria, 23 patients with microalbuminuria and 21 patients with macroalbuminuria. Serum concentrations of ANP and cytokines were measured using enzyme-linked immunosorbent assay (ELISA) kits. The correlations between ANP and clinical variables were analyzed. Multiple linear regression and logistic regression models were constructed to test the associations between ANP and the severity and presence of albuminuria.

**Results:**

The macroalbuminuria patients exhibited higher plasma levels of ANP, TNF-α, IL-6, and ADP; higher serum creatinine (Cr) and blood urea nitrogen (BUN); and longer duration of diabetes mellitus (DM) than the patients with normoalbuminuria and microalbuminuria. Plasma ANP level was significantly associated with TNF-α (*r* = 0.876, *p* < 0.001), IL-6 (*r* = 0.816, *p* < 0.001) and ADP (r = 0.772, *p* < 0.001), independent of the duration of DM or the BUN concentration.

**Conclusion:**

ANP is higher in type 2 diabetes mellitus nephropathy subjects, especially those who have macroalbuminuria, which is associated with compensatory responses to inflammation.

## Background

Diabetic nephropathy (DN) is an important microvascular-related diabetes complication that accounts for at least 50% of end-stage renal disease (ESRD) [1, 2]. Early diagnosis of DN prevents its progression to diabetic kidney disease.

By analyzing fundamental cytokines, we may find markers that can identify T2DM and molecules that can be targeted for treatment, particularly those predictive of DN. Atrial natriuretic factor (ANP) refers to a member of a family of cardiac- and vascular-derived hormones primarily secreted from the atrial wall involved in the control of increased volume or elevated blood pressure [3]. ANP has been recently identified as acting on blood vessels and the adrenal gland, exhibiting natriuretic and vasodilating properties and critically controlling volume homeostasis and blood pressure [4, 5]. Several analyses have demonstrated elevated serum levels of natriuretic peptide in patients developing chronic kidney disease (CKD) under the complication of impaired renal function. In those reports, the mentioned peptides seem to be markers of renal function reduction or CKD progression [6, 7]. ANP also participates in adipose metabolic pathways [8]. Adipose tissue is an important endocrine tissue whose adipocytes and stromal cells secrete numerous biologically active proteins and cytokines, respectively [9]. Adiponectin (ADP) is a protein secreted only by adipose tissues and appears to act as a hormone that downregulates inflammatory responses in vitro. Likewise, decreased ADP is associated with T2DM and its relevant macrovascular complications, as well as insulin resistance [10, 11]. However, recent studies have pointed out that the levels of urinary or serum ADP are elevated in both primary nephrotic syndrome and DN [12, 13]. ANP may regulate adipocyte metabolism, including ADP, through lipolysis, lipid oxidation, and adipocyte browning, and it may directly influence macrophages [14–16]. Several inflammatory cytokines (e.g., interleukin-6 (IL-6) and tumor necrosis factor-α (TNF-α)) play roles in glomerular and vascular endothelial cell damage and affect the level of urinary albumin in T2DM patients.

No data regarding the relationship between ANP levels and DN are available. The present study aimed to explore the association between ANP and DN. The effect exerted by ANP on T2DM albuminuria patients was analyzed. Moreover, the concentrations of ADP along with TNF-α and IL-6 were measured to elucidate the interaction, if any, of the mentioned cytokines and ANP in patients stratified by albuminuria degree.

## Methods

### Subjects

Eighty-one patients diagnosed with T2DM were recruited from the Department of Endocrinology of Nanjing First Hospital, Nanjing Medical University, from May to August 2016. The study complied with the Helsinki Declaration for the investigation of human subjects. The Ethics Committee of Nanjing First Hospital approved this study. Informed consent was obtained from each participant enrolled in the study. The subjects were divided into 3 groups according to the guidelines for chronic kidney disease [17]: 21 with macroalbuminuria (Macro-MA group), 23 with microalbuminuria (Micro-MA group) and 37 with normoalbuminuria (Nor-MA group). Macroalbuminuria, microalbuminuria and normoalbuminuria were defined as urine albumin excretion (UAE) > 300 mg/24 h, 30–300 mg/24 h and < 30 mg/24 h [17], respectively.

The exclusion criteria were (1) history of overt cerebrovascular disease (including any degree of cardiac insufficiency, angina pectoris and stroke); (2) severe, nonregulated high blood pressure, defined as systolic blood pressure > 160 mmHg/diastolic blood pressure > 100 mmHg; (3) severe dyslipidemia (total cholesterol > 400 mg/d; (4) urinary tract infection or any other acute inflammation or infection; (5) kidney diseases besides DN or the use of anticonvulsant drugs, anti-inflammatory drugs or nephrotoxic drugs; (6) diagnosed liver failure or malignancy under the previous diagnosis; (7) current pregnancy or lactation; (8) positivity for islet cell autoantibodies, indicating the possibility of type 1 diabetes mellitus; (9) current treatment with thiazolidinediones, which are known to increase the plasma ADP level.

### Clinical measurements

Physical examinations were performed, which included measurements of blood pressure, height, weight and other anthropometric parameters. Samples of venous blood were drawn from all patients after an 8-h fast before and after treatment. Biochemical and hematological properties were determined with routine technologies in one analyzing instrument. Blood was collected into 4 ml EDTA-containing tubes and centrifuged for 30 min at 3000 rpm for 10 min. Serum samples were stored as aliquots with no preservatives at − 80 °C for 3 months on average until the cytokine study.

### Assessment of circulating ANP and cytokines

Serum ANP (catalog number bsk00431, Bioss, Burlington, ON, Canada), ADP (catalog number bsk00199, Bioss, Burlington, ON, Canada), IL-6 (catalog number bsk00040, Bioss, Burlington, ON, Canada) and TNF-α (catalog number bsk00162, Bioss, Burlington, ON, Canada) levels were measured by using commercially available human ELISA kits according to the manufacturer’s instructions. The detection limits for ANP, ADP, IL-6 and TNF-α were 10 ng/L, 30 μg/L, 2 ng/L and 8 pg/L, respectively. The intratest and intertest coefficients of variation were 5 and 10%, respectively.

Body mass index (BMI) was calculated as weight (kg)/square of height (m).

### Statistical analysis

SPSS (Statistical Package for Social Sciences) version 20.0 for Windows was used for statistical analysis. For continuous, normally distributed data, mean ± SD values are reported; otherwise, the median (interquartile range) is reported. To assess correlations of categorical variables with the Macro-MA and Nor-MA groups, the chi-square test was used. ANOVA compared variables between 3 groups. The Kruskal-Wallis test was used to compare three groups on nonnormal variables. Correlations were tested by conducting regression analysis. To determine the relationship between numerical variables for normally distributed groups, Pearson’s correlation coefficient was calculated, and Spearman’s test was used for abnormally distributed groups. Logistic regression analysis was conducted to assess the associations between ANP level and the other parameters assessed. Two-tailed *p* values < 0.05 were considered significant.

## Results

According to Table 1, the individuals in the 3 groups were well matched for age and sex composition. SBP was significantly higher in the Macro-MA group than in the other 2 groups, while DBP was comparable between the 3 groups. No differences were identified in alanine transaminase (ALT), aspartate transaminase (AST), total cholesterol (TC), triglycerides (TG), high-density lipoprotein cholesterol (HDL-C), low-density lipoprotein cholesterol (LDL-C), Na, K or uric acid (UA) between the 3 groups. Naturally, the glycosylated hemoglobin (HbA1c) levels of all patients were above the normal range, but HbA1c was lower in the Macro-MA group than in the other two groups (*p* < 0.05). Glucose in the Macro-MA group was 6.35 (5.09, 8.08) mmol/L, which was distinctly lower than that in the other two groups. Macroalbuminuria patients showed markedly higher durations of DM, Cl, serum creatinine, and urea but lower plasma albumin levels (*p* < 0.05 in all comparisons).

**Table 1:** General clinical profiles between 3 groups

Variable	Nor-MA	Micro-MA	Macro-MA	*p* value
N	37	23	21	–
Men (%)	23 (62.2)	11 (47.82)	11 (52.38)	0.533^a^
Age (years)	61.32 (11.69)	66.17 (13.41)	66.10 (8.68)	0.181^b^
BMI (kg/m^2^)	23.24 (20.85,26.86)	24.24 (21.97,26.67)	24.78 (22.95,27.24)	0.709^b^
Duration (years)	3.40 (3.49)	10.13 (6.61)	16.52 (7.90)**	< 0.001^a^
SBP (mmHg)	127 (13)	133 (17)	139 (17)**	0.025^a^
DBP (mmHg)	78 (7)	77 (8)	79 (8)	0.522^a^
UAE (mg/24 h)	11.94 (7.44,21.09)	96.90 (54.78,168.66)	1155.77 (854.69,1575.00)**	< 0.001^a^
HbA1c (%)	8.50 (7.00,10.55)	9.10 (7.00,10.20)	7.10 (6.15,8.20)*	0.018^b^
ALT (IU/L)	23.16 (11.78)	30.09 (40.82)	19.19 (9.14)	0.299^a^
AST (IU/L)	27.57 (19.13)	30.83 (26.49)	20.82 (13.53)	0.256^a^
TC (mmol/L)	4.78 (3.80,5.35)	4.45 (3.22,5.53)	5.26 (3.84,6.14)	0.286^b^
TG (mmol/L)	1.88 (1.66)	2.18 (2.05)	2.71 (2.43)	0.317^a^
HDL-C (mmol/L)	1.15 (0.98,1.35)	1.05 (0.92,1.36)	1.05 (0.87,1.24)	0.421^b^
LDL-C (mmol/L)	2.54 (1.91,3.03)	2.27 (1.37,2.84)	2.95 (1.80,3.50)	0.229^b^
BUN (mmol/L)	6.02 (1.26)	7.28 (3.84)	11.26 (6.41)**	< 0.001^a^
Cr (umol/L)	68.34 (21.80)	86.73 (57.11)	175.38 (109.76)**	< 0.001^a^
Na (mmol/L)	141.80 (139.45,142.85)	140.70 (139.60,142.60)	141.70 (139.10,144.15)	0.610^b^
K (mmol/L)	3.83 (3.60,3.99)	3.87 (3.56,4.30)	3.77 (3.34,4.36)	0.728^b^
Cl (mmol/L)	101.90 (100.95,102.30)	102.80 (101.10,104.90)	104.90 (102.15,107.35)**	< 0.001^b^
UA (umol/L)	353.00 (251.50,428.00)	298.00 (263.00,465.00)	355.00 (306.00,476.00)	0.415^b^
ALB(g/L)	40.50 (38.60,40.75)	41.40 (36.50,43.60)	34.80 (30.70,38.05)**	< 0.001^b^

*BMI* body mass index, *SBP* systolic blood pressure, *DBP* diastolic blood pressure, *UAE* urinary albumin excretion, *HbA1c* glycosylated hemoglobin, *ALT* alanine transarninase, *AST* aspartate transaminase, *HDL-C* high-density lipoprotein cholesterol, *LDL-C* low-density lipoprotein cholesterol, *BUN* blood urea nitrogen, *Cr* plasma creatinine, *TC* total cholesterol, *TG* triglycerides, *UA* uric acid, *ALB* plasma albumin

Continuous variables were expressed as mean (standard deviation) and non-normally distributed variables were expressed as median (interquartile range). An ANOVA test was used to to compare differences of mean between 3 groups (^a^). The Kruskal-Wallis test was used to compare differences of mean between 3 groups with normal distribution (^b^)

*p < 0.05 compared with control group,**p < 0.01 compared with control group

The serum ANP level in the Macro-MA group was significantly higher than that in the other two groups (11.25 ± 4.05 ng/L and 13.58 ± 6.43 ng/L in normoalbuminuria and microalbuminuria, respectively). Interestingly, compared to the patients with normoalbuminuria, the macroalbuminuria patients had higher serum ADP (*p* < 0.001). Furthermore, the levels of TNF-α and IL-6 increased as nephropathy progressed, with median serum TNF-α levels of 13.49 pg/mL in the normoalbuminuric group, 15.22 pg/mL in the Micro-MA group, and 18.28 pg/mL in the Macro-MA group. The median serum levels of IL-6 were 2.13 ng/L in the normoalbuminuric group, 2.20 ng/L in the Micro-MA group, and 3.21 ng/L in the Macro-MA group (Table 2).

**Table 2:** Plasma cytokines levels of patients according to the albuminuria categories

Variable	Nor-MA	Micro-MA	Macro-MA
ANF (ng/L)	11.25 (4.05)	13.58 (6.43)	19.20 (13.43)**
TNF-α (pg/mL)	13.49 (4.67)	15.22 (7.57)	18.28 (13.50)*
ADP (ug/L)	4.96 (2.52)	5.55 (2.74)	7.96 (6.16)**
IL-6(ng/L)	2.13 (1.18)	2.20 (1.35)	3.21 (2.95)*

*ANP* atrial natriuretic peptide, *ADP* adiponection, *IL-6* interleukin-6, *TNF-α* tumor necrosis factor-α

*p < 0.05 compared with control group,**p < 0.01 compared with control group

**Fig. 1:**
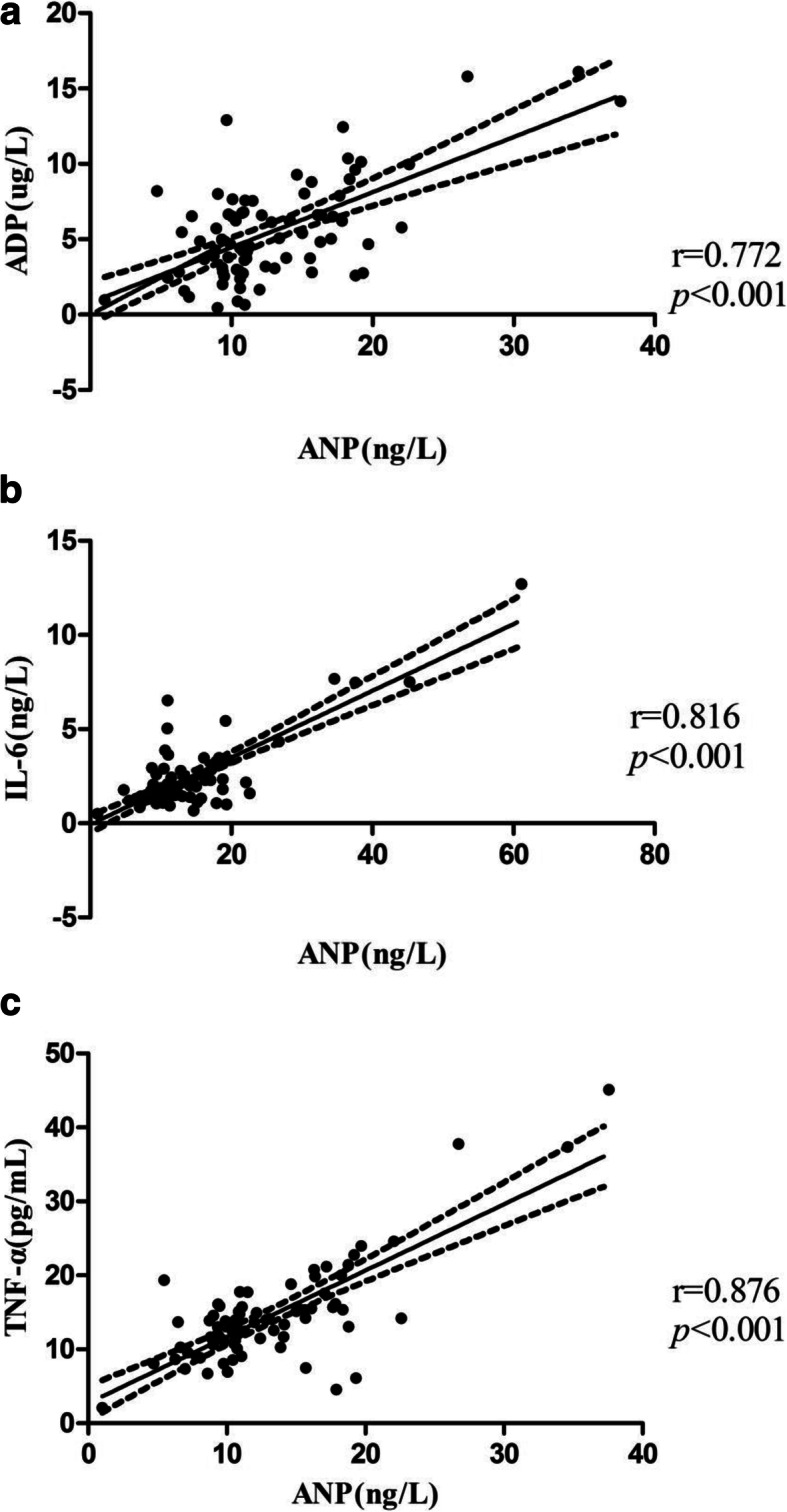
**a** Correlation between ANP and serum ADP in patients with T2DM. **b** Correlation between ANP and serum IL-6 in patients with T2DM. **c** Correlation between ANP and serum TNF-α in patients with T2DM

The above variables were assessed by multivariate statistical analysis (Table 3). In the entire sample, Pearson’s or Spearman’s linear correlation test revealed a positive relationship of ANP with UAE (*r* = 0.235, *p* = 0.035), BUN (*r* = 0.235, *p* = 0.034), and plasma ADP (*r* = 0.772, *p* < 0.001) (Fig. 1a), IL-6 (*r* = 0.816, *p* < 0.001) (Fig. 1b) and TNF-α (*r* = 0.876, *p* < 0.001) (Fig. 1c). A correlation was noted between ANP and the duration of DM in simple linear regression. There was a negative relationship between ANP and plasma albumin (*r* = − 0.267, *p* = 0.016). There was no noticeable correlation between ANP and any other variable: BP, lipid levels, glucose, liver enzymes and urea (Table 3). In univariate analysis, all the parameters listed in Table 4 displayed associations with the DN development process. In Model 1, because the duration of diabetes was an independent risk factor, we excluded it from the analysis, and all the parameters included in this model were significant factors for DN development. Model 2 included the duration of diabetes, and the results showed that both UAE and BUN were not significant predictors, while plasma ANP and DM duration were correlated with each other. The duration of DM was a powerful confounder in the relationship between ANP and DN development. Furthermore, ADP, TNF-α and IL-6 still correlated independently with ANP (*p* < 0.05) after adjustment for the duration of diabetes and BUN.

**Table 3:** Correlation analysis between serum ANP and the clinical parameters

Variable	r	*p*
Age	0.034	0.760^a^
BMI	0.109	0.334^b^
Duration	0.285	0.010 ^a^
SBP	0.066	0.559^a^
DBP	0.087	0.442^a^
HbA1c	−0.003	0.978^b^
UAE	0.235	0.035^b^
TC	0.072	0.552^b^
TG	0.106	0.346^a^
HDL-C	−0.023	0.838^b^
LDL-C	0.034	0.764^b^
BUN	0.235	0.034^a^
Cr	0.198	0.076^a^
UA	0.123	0.274^b^
ALB	−0.267	0.016^b^
ADP	0.772	< 0.001^a^
IL-6	0.816	< 0.001^a^
TNF-α	0.876	< 0.001^a^

*BMI* body mass index, *SBP* systolic blood pressure, *DBP* diastolic blood pressure, *UAE* urinary albumin excretion, *HbA1c* glycosylated hemoglobin, *ALT* alanine transarninase, *AST* aspartate transaminase, *HDL* high-density lipoprotein cholesterol, *LDL* low-density lipoprotein cholesterol, *Cr* plasma creatinine, *BUN* blood urea nitrogen, *TC* total cholesterol, *TG* triglycerides, *UA* uric acid, *ALB* plasma albumin, *ANP* atrial natriuretic peptide, *ADP* adiponection, *IL-6* interleukin-6, *TNF-α* tumor necrosis factor-α

^a^ Pearson test

^b^ Spearman test

**Table 4:** Multiple regression model for the determinants of serum ANP

Independent variable	Model 1					Model 2				
	β	SE	Wald	*p*	OR	β	SE	Wald	*p*	OR
UAE (mg/24 h)	0.150	0.211	8.227	0.033	1.92 (1.37–2.27)	0.059	0.509	9.231	0.450	1.12 (1.03–2.32)
TNF-α (pg/mL)	0.528	0.254	9.017	< 0.001	2.23 (1.13–2.43)	0.519	0.673	12.187	< 0.001	2.01 (1.45–3.61)
ADP (ug/L)	0.233	0.365	7.157	0.001	2.07 (1.14–2.87)	0.224	0.396	10.172	0.001	2.31 (2.11–3.12)
IL-6 (ng/L)	0.255	0.373	7.651	0.006	2.62 (1.42–3.19)	0.233	0.523	7.894	0.005	2.89 (2.71–3.58)
BUN (mmol/L)	0.127	0.403	9.187	0.032	1.93 (1.60–2.45)	0.041	0.652	8.921	0.592	1.16 (1.11–2.45)

*UAE* urinary albumin excretion, *ANP* atrial natriuretic peptide, *ADP* adiponection, *IL-6* interleukin-6, *TNF-α* tumor necrosis factor-α, *BUN* blood urea nitrogen

Model 1 is unadjusted for duration of diabetes

Model 2 is adjusted for duration of diabetes

## Discussion

In this study of 81 T2DM participants, 44 had albuminuria and 40 did not. The blood inflammatory cytokines and ANP and the albuminuria levels were measured. Post hoc analysis showed as follows: (1) serum ANP was higher in the DN group than in the control (T2DM) group [18–20]; (2) serum ANP was higher in the macroalbuminuria stage of DN; (3) serum ADP, TNF-α, and IL-6 levels were increased in the presence of DN; (4) serum ANP level was significantly positively correlated with ADP, TNF-α, and IL-6 levels; (5) serum ANP was positively correlated with DN stage after adjusting for the risk factors; and (6) ANP was level demonstrated to be an independent risk factor for DN.

ANP is a cardiac hormone that provides negative feedback under elevated blood pressure and fluid volume [3]. Robert et al. [21] showed that ANP may well improve intravascular protein glomerular permeability in the glomerulus when synthetic human ANP is infused in patients with nephrotic syndrome. ANP dilates afferent arterioles and constricts efferent arterioles, producing an increase in glomerular capillary pressure and leading to a rise in the fractional excretion of proteins [22]. For the foregoing reasons, ANP may induce microalbuminuria. According to Desai et al. [23], the higher the plasma level of ANP is, the more rapidly renal function deteriorates. We did not expect ANP to be elevated during DN development, though there are several possible explanations for this finding. First, reduction in renal function with disease course may elevate fluid volume, and higher left atrial pressure induces greater secretion of ANP. Second, one major metabolic pathway of ANP involves binding to clearance receptors (NPR-C) [24]. As revealed in one recent study, the number of NPR-C proteins on platelets in elderly subjects decreased, which could be associated with a reduction in the synthesis of this receptor or delayed recycling to the cell surface [25]. Third, ANP is downregulated by neutral endopeptidase (NEP), an enzyme expressed in the kidney. NEP expression in renal tissue was downregulated among chronic renal failure patients. These changes eventually result in decreased clearance of ANP from the circulation. Thus, ANP may participate in DN development, but the precise mechanisms remain unclear.

ANP is capable of regulating lipolysis and lipid mobilization in humans. Moreover, it might regulate adipose tissue inflammation by regulating the secretion of inflammatory cytokines [26, 27]. The elevated levels of TNF-α, IL-6 and ADP here showed strong positive correlations with ANP concentration and the degree of albuminuria, in line with existing studies [18, 19]. As revealed in one study, adipocytes probably express all components of the ANP signaling pathway [16]. ANP induces the secretion of IL-6 and TNF-α via the guanylate cyclase-coupled A receptor (NPR-A) in macrophages [28]. Activation of the NPR-A receptor stimulates a rise in cGMP levels, activating protein kinase G; as a result, lipolysis is stimulated, to produce IL-6 and TNF-α. ADP, a “conducive” adipokine that reduces inflammation, is reduced in diabetes, and high ADP levels are associated with a lower risk of developing T2DM [29–31]. Nevertheless, ADP concentrations are higher in patients with high albuminuria, especially in Macro-MA subjects, and positively predict the prognosis of this disease [32]. ADP is known to act via AMP-activated protein kinase, as does ANP in adipocytes [33]. A similar effect was also indicated in healthy people, in which ANP raises ADP in a dose-dependent manner [34]. This phenomenon is in line with the possible mechanism by which ANP directly upregulates ADP and thereby counteracts ANP-induced lipolysis. One small clinical study has reported that decompensated heart failure patients who had undergone therapeutic ANP infusions had increased plasma levels of total and high-molecular-weight ADP [35]. As reported by recent studies, during DN development, increased production and excretion of inflammatory cytokines result in aggravated glomerular hypertrophy and disappearance of podocytes, which are activated and contribute to deteriorating kidney function [36]. ANP may promote the progression of DN by stimulating the secretion of inflammatory cytokines. Further investigation of ANP receptor antagonists or enzyme inhibitors can significantly improve and postpone the progression of DN.

Patients treated with thiazolidinediones, which are known to partly increase the plasma ADP level, as well as those treated with thiazolidinediones, anticonvulsant drugs, anti-inflammatory drugs or nephrotoxic drugs, were excluded because these drugs affect the level of inflammation detected.

Notably, HbA1c levels were decreased more in the macro-MA group than in the other 2 groups. This could be explained by the fact that patients with a longer duration of DM and diabetic complications are more diligent about managing their blood glucose. In addition, as suggested in a previous study, ANP may impact the etiology of diabetes primarily by inhibiting glucagon secretion [37] and the enzyme insulinase produced in the kidneys. Moreover, Sabrina U et al. [38] isolated pancreatic islets from adult mice and showed that ANP enhanced insulin secretion under the stimulation of glucose and triggered β-cell growth.

This study has some limitations. First, the cross-sectional design for the baseline group analysis and the restricted size of each group are likely to affect the result. Randomized studies with larger samples will be required for more in-depth exploration. Second, we cannot completely exclude the possibility that ANP influenced the clearance of renal or hepatic cytokines. Finally, only plasma cytokines were measured, but measuring ANP and other cytokines in urine might help in the interpretation of the results.

## Conclusion

In summary, increased circulating concentrations of plasma ANP, which were correlated with a longer duration of DM, could precede the development of type 2 diabetic kidney disease with no noticeable cardiovascular disease, revealing one likely effect exerted by such bioactive peptides that could be useful in monitoring the DN development process in the early phase. Further investigations on the molecular mechanism of ANP in the kidney and of inflammatory cytokines should be performed to prevent and delay renal disease development in T2DM patients.

## Data Availability

Our data will not be shared due to it was involved in individual privacy and needed further study.

## References

[1] Kausik U, Julia BL (2018). Update on diabetic nephropathy: Core curriculum 2018. Am J Kidney Dis.

[2] Annabelle MW, Søren TK, Mark EC (2019). Diabetic nephropathy: an insight into molecular mechanisms and emerging therapies. Expert Opin Ther Targets.

[3] Rui T, You MA, Hye YK, Yun JL, Kyung WC, Dae GK, Ho SL (2018). Atrial secretion of ANP is suppressed in renovascular hypertension: shifting of ANP secretion from atria to the left ventricle. Am J Physiol Heart Circ Physiol.

[4] Chiaki NO, Kenji K, Naoto M (2017). Three molecular forms of atrial natriuretic peptides: quantitative analysis and biological characterization. J Pept Sci.

[5] Tomoko I, John CB (2017). Atrial natriuretic peptide - old but new therapeutic in cardiovascular diseases. Circ J.

[6] Franziska T, Qingyu W (2015). ANP-induced signaling cascade and its implications in renal pathophysiology. Am J Physiol Renal Physiol.

[7] Spanaus KS, Kronenberg F, Ritz E, Schlapbach R, Fliser D, Hersberger M, Kollerits B, Konig P, von Eckardstein A (2007). Mild-to-moderate kidney disease study G: B-type natriuretic peptide concentrations predict the progression of nondiabetic chronic kidney disease: the mild-to-moderate kidney disease study. Clin Chem.

[8] Wang Y, Xu L, Yuan L, Li D, Zhang Y, Zheng R, Liu C, Feng X, Li Q, Li Q (2016). Sodium-glucose co-transporter-2 inhibitors suppress atrial natriuretic peptide secretion in patients with newly diagnosed type 2 diabetes. Diabetic Med.

[9] Robert W (2018). O'R. Adipose tissue and the physiologic underpinnings of metabolic disease. Surg Obes Relat Dis.

[10] Ravindran J, Rajeswari R, Sugapriya D (2018). Emerging Role of Adipocytokines in Type 2 Diabetes as Mediators of Insulin Resistance and Cardiovascular Disease. Can J Diabetes.

[11] Ze FC, Yulan B, Xin YL, Qian QL, Zheng W, Yuan FL, Sheng ZH, Yun KY, Zeng NM (2020). Effects of Adiponectin on T2DM and glucose homeostasis: a Mendelian randomization study. Diabetes Metab Syndr Obes.

[12] Jun YL, Jae WY, Byoung GH, Seung OC, Jae SK (2019). Adiponectin for the treatment of diabetic nephropathy. Korean J Intern Med.

[13] Wei Y, Qian OY (2019). Adiponectin improves diabetic nephropathy by inhibiting necrotic apoptosis. Arch Med Sci.

[14] Andrea T, Federica V, Miriam P, Marco G, Laura S, Roberto B, Lucia F (2019). Adipose tissue, obesity and Adiponectin: role in endocrine Cancer risk. Int J Mol Sci.

[15] Bordicchia M, Liu D, Amri EZ, Ailhaud G, Dessi-Fulgheri P, Zhang C, Takahashi N, Sarzani R, Collins S (2012). Cardiac natriuretic peptides act via p38 MAPK to induce the brown fat thermogenic program in mouse and human adipocytes. J Clin Invest.

[16] Moro C, Klimcakova E, Lolmede K, Berlan M, Lafontan M, Stich V, Bouloumie A, Galitzky J, Arner P, Langin D (2007). Atrial natriuretic peptide inhibits the production of adipokines and cytokines linked to inflammation and insulin resistance in human subcutaneous adipose tissue. Diabetologia.

[17] Paul ES, Adeera L (2013). Valuation and management of chronic kidney disease: synopsis of the kidney disease: improving global outcomes 2012 clinical practice guideline. Ann Intern Med.

[18] Lampropoulou IT, Stangou M, Papagianni A, Didangelos T, Iliadis F, Efstratiadis G (2014). TNF-alpha and microalbuminuria in patients with type 2 diabetes mellitus. J Diabetes Res.

[19] Bin C, Meiyan W, Chongsen Z, Yan HL, Zhong GX (2019). Association between IL-6 polymorphisms and diabetic nephropathy risk: a Meta-analysis. Am J Med Sci.

[20] Ming Z, Jungang H (2018). Dendrobium Officinale Kimura et Migo ameliorates insulin resistance in rats with diabetic nephropathy. Med Sci Monit Basic Res.

[21] Zietse R, Schalekamp MA (1988). Effect of synthetic human atrial natriuretic peptide (102-126) in nephrotic syndrome. Kidney Int.

[22] Franziska T, Qing YW (2015). ANP-induced signaling cascade and its implications in renal. Am J Physiol Renal Physiol.

[23] Desai AS, Toto R, Jarolim P, Uno H, Eckardt KU, Kewalramani R, Levey AS, Lewis EF, McMurray JJ, Parving HH (2011). Association between cardiac biomarkers and the development of ESRD in patients with type 2 diabetes mellitus, anemia, and CKD. Am J Kidney Dis.

[24] Yang G, Donna T, Jie X, David FL, John AM, Danielle BC, Charles EM, Yuping W (2018). Aberrant pro-atrial natriuretic peptide/corin/natriuretic peptide receptor signaling is present in maternal vascular endothelium in preeclampsia. Pregnancy Hypertens.

[25] Motoyuki I, Seiji M, Takeshi O, Jun S, Shin YK, Ryui CA, Mitsuo M (2008). Arterial stiffness, physical activity, and atrial natriuretic peptide gene polymorphism in older subjects. Hypertens Res.

[26] Chen C, Hui XL, Cheng CT, Dou DT, Yong XZ, Xia L, Wen XS, Lin LW, Lina L, Jia L, Cheng HW, Qiu YC, Yi MD, Qing KW, Xiang R (2019). Mutation in NPPA causes atrial fibrillation by activating inflammation and cardiac fibrosis in a knock-in rat model. FASEB J.

[27] Li BM, Jin RZ, Bi WM, Chang MW, Ya BS, Meng Z, Shao KL, Xu DX, Cong YW (2015). ANP/NPRA signaling preferentially mediates Th2 responses in favor of pathological processes during the course of acute allergic asthma. Int J Clin Exp Med.

[28] Vanessa V, David S, Khanh L, Bruce KW, Baljit S (2019). Mouse model to study pulmonary intravascular macrophage recruitment and lung inflammation in acute necrotizing pancreatitis. Cell Tissue Res.

[29] Gupta C, Bubber P, Fahim M, Saidullah B, Omanwar S (2020). Adiponectin in onset and progression of T2DM with cardiac dysfunction in rats. Hum Exp Toxicol.

[30] Chen XL, Xiu F, Qi L, Ying W, Qian L, Jian MH (2016). Adiponectin, TNF-α and inflammatory cytokines and risk of type 2 diabetes: a systematic review and meta-analysis. Cytokin.

[31] Wei L, Xiang HZ, Yu FL, Si MZ, Xiao LC, Rui Z, Si QG, Xue YH, Linong J (2020). Serum leptin, resistin, and adiponectin levels in obese and non-obese patients with newly diagnosed type 2 diabetes mellitus: a population-based study. Medicine (Baltimore).

[32] Ran J, Xiong X, Liu W, Guo S, Li Q, Zhang R, Lao G (2010). Increased plasma adiponectin closely associates with vascular endothelial dysfunction in type 2 diabetic patients with diabetic nephropathy. Diabetes Res Clin Pract.

[33] Souza SC, Chau MD, Yang Q, Gauthier MS, Clairmont KB, Wu Z, Gromada J, Dole WP (2011). Atrial natriuretic peptide regulates lipid mobilization and oxygen consumption in human adipocytes by activating AMPK. Biochem Biophys Res Commun.

[34] Birkenfeld AL, Boschmann M, Engeli S, Moro C, Arafat AM, Luft FC, Jordan J (2012). Atrial natriuretic peptide and adiponectin interactions in man. PLoS One.

[35] Tanaka T, Tsutamoto T, Sakai H, Nishiyama K, Fujii M, Yamamoto T, Horie M (2008). Effect of atrial natriuretic peptide on adiponectin in patients with heart failure. Eur J Heart Fail.

[36] Barutta F, Bruno G, Grimaldi S, Gruden G (2015). Inflammation in diabetic nephropathy: moving toward clinical biomarkers and targets for treatment. Endocrine.

[37] Verspohl EJ, Bernemann IK (1996). Atrial natriuretic peptide ()-induced inhibition of glucagon secretion: mechanism of action in isolated rat pancreatic islets. Peptides.

[38] Sabrina U, Julia K, Jelena S, Martina D, Peter KD, Gisela D (2017). Atrial natriuretic peptide affects stimulus-secretion coupling of pancreatic β-cells. Diabetes.

